# Supplementary material for: Content-rich biological network constructed by mining PubMed abstracts
Source: BMC Bioinformatics. 2004 Oct 8;5:147. doi: 10.1186/1471-2105-5-147 (PMC528731; doi:10.1186/1471-2105-5-147)
Supplement: Additional File 2 — The original results of the above study (non-essential files are deleted to keep the file size under the limit set by BMC bioinformatics). [file 1471-2105-5-147-S2.bz2 › chilibotAdditionalFile2/dip20/351ID8663118E4684/html/IL13_IL13RA2.html]

 


 **IL13** and **IL13RA2** 
  
Found 82 abstracts in PubMed, retrieved 20.  
 

 What does Google say? 
 PDF only 
| .edu only 

---

**Interactive relationship** (e.g. stimulation, inhibition, etc)

**Neutral relationship**- Interleukin 13  [ **IL13** ]  receptor IL 13R  [ **IL13RA2** ]  targeted cytotoxin,  **IL13**  PE38QQR, composed of IL 13  [ **IL13** ]  and a mutated form of Pseudomonas exotoxin PE, is found to be highly and specifically cytotoxic to human solid cancer cell lines.  Ref: 11862421 Cancer Immunol Immunother, 2002
- Our third generation experiments suggest that this approach is feasible for clinical situations as intratumor administration of plasmid carrying the IL 13R  [ **IL13RA2** ]  alpha2 chain gene sensitized these tumors to systemic or regional  **IL13**  PE therapy.  Ref: 12187325 Anticancer Drugs, 2002
- RT PCR showed that each culture expressed IL 4R alpha, common gamma chain, and IL 13R  [ **IL13RA2** ]  alpha 1, as well as IL 13R  [ **IL13RA2** ]  alpha 2, which negatively regulates IL 13  [ **IL13** ]  signaling.  Ref: 12077271 J Immunol, 2002
- Thus, IL 13  [ **IL13** ]  cytotoxin may be a useful agent for high IL 13R  [ **IL13RA2** ]  expressing SCCHN.  Ref: 12060640 Clin Cancer Res, 2002
- Acquisition of sensitivity to  **IL13**  PE was observed both in vitro and in vivo when IL 13R  [ **IL13RA2** ]  alpha2 transfected human tumor cells were implanted in immunodeficient animals followed by systemic or regional  **IL13**  PE therapy.  Ref: 12187325 Anticancer Drugs, 2002
- IL 13  [ **IL13** ]  cytotoxin, composed of IL 13  [ **IL13** ]  and a truncated form of Pseudomonas exotoxin, targets IL 13R  [ **IL13RA2** ]  overexpressing tumor cell lines in vitro and in vivo.  Ref: 12455052 Int J Cancer, 2003
- BACKGROUND IL 13  [ **IL13** ]  receptor IL 13R  [ **IL13RA2** ]  targeted cytotoxin,  **IL13**  PE38QQR, has been shown to have very potent anti tumor activity to IL 13R  [ **IL13RA2** ]  expressing head and neck tumor cells in vitro and in vivo.  Ref: 12435859 Mol Med, 2002
- In contrast, normal human astrocytes that did not express the IL 13R  [ **IL13RA2** ]  alpha 2 gene efficiently induced Stat6 activation in response to both IL 4 and IL 13  [ **IL13** ] .  Ref: 11861389 Cancer Res, 2002
- This was in part because of the expression of a high affinity IL 13  [ **IL13** ]  binding transmembrane protein IL 13R  [ **IL13RA2** ]  alpha 2 that inhibited IL 13  [ **IL13** ]  mediated Stat6 activation by acting as a decoy receptor.  Ref: 11861389 Cancer Res, 2002

**Non-interactive relationship** (e.g. studied together, co-existance, homology, etc.)

- We have previously reported that interleukin 13  [ **IL13** ]  receptors IL 13R  [ **IL13RA2** ]  are constitutively overexpressed on a majority of human malignant glioma cell lines and primary cell cultures.  Ref: 12481422 Mol Cancer Ther, 2002
- IL 13R  [ **IL13RA2** ]  alpha 2, a decoy receptor for IL 13  [ **IL13** ]  acts as an inhibitor of IL 4 dependent signal transduction in glioblastoma cells.  Ref: 11861389 Cancer Res, 2002
- it also acts as the signaling chain for IL 13  [ **IL13** ] , but in this case the IL 13R  [ **IL13RA2** ]  alpha 1 chain conveys the bulk of the cytokine specificity.  Ref: 12165974 Roum Arch Microbiol Immunol< MedlineTA>Roum Arch Microbiol Immunol, 2002
- Although some prostate cancer cell lines express functional IL 13R  [ **IL13RA2** ] , they are not highly sensitive to IL 13  [ **IL13** ]  cytotoxin.  Ref: 11773976 Cancer Gene Ther, 2001
- To study the function of IL 13R  [ **IL13RA2** ]  on brain tumor cells, we investigated the regulation of adhesion molecules by IL 13  [ **IL13** ]  as assessed by flow cytometric analysis.  Ref: 11939409 Oncol Res, 2000
- These receptors are composed of IL 13  [ **IL13** ]  receptor IL 13R  [ **IL13RA2** ]  alpha1, IL 13Ralpha2, and IL 4Ralpha chains.  Ref: 11939409 Oncol Res, 2000
- We have demonstrated that when interleukin IL 13  [ **IL13** ]  receptor IL 13R  [ **IL13RA2** ]  alpha2 chain, one of the two known IL 13  [ **IL13** ]  binding proteins, is introduced into cancer cells that do not express this chain the cells acquire extreme sensitivity to a chimeric fusion cytotoxin composed of IL 13  [ **IL13** ]  and a mutated form of Pseudomonas exotoxin  **IL13**  PE.  Ref: 12187325 Anticancer Drugs, 2002
- IL 13  [ **IL13** ]  and IL 13R  [ **IL13RA2** ]  alpha 1, the IL 13  [ **IL13** ]  specific receptor chain, are frequently expressed by HL derived cell lines and by RS cells from biopsy material of tissues involved by HL.  Ref: 11815733 Int Arch Allergy Immunol, 2001
- Coexpression of IL 13  [ **IL13** ] , IL 13R  [ **IL13RA2** ]  alpha 1 and P STAT6 is uncommon in non Hodgkin lymphomas.  Ref: 11815733 Int Arch Allergy Immunol, 2001
- Interleukin 13  [ **IL13** ]  receptor IL 13R  [ **IL13RA2** ]  alpha2 chain plays a key role in ligand binding and internalization.  Ref: 12808442 Gene Ther, 2003
- This was followed by successfully treating xenograft tumor bearing nude mice with IL 13R  [ **IL13RA2** ]  directed cytotoxin  **IL13**  PE38QQR.  Ref: 12471149 J Immunol, 2002
- Here, we report that Jak2 is associated with IL 4R alpha, and Tyk2 is associated with the IL 13R  [ **IL13RA2** ]  alpha 1 component of the IL 13  [ **IL13** ]  receptor complex.  Ref: 12223527 J Leukoc Biol, 2002
- IL 13  [ **IL13** ]  and the IL 13  [ **IL13** ]  specific receptor chain IL 13R  [ **IL13RA2** ]  alpha1 are frequently expressed in cHL derived cell lines and in RS cells from biopsies of cHL tissues.  Ref: 12152987 Leuk Lymphoma, 2002
- Transient expression of the IL 13R  [ **IL13RA2** ]  alpha 2 transgene in nonexpressing heterologous cells inhibited not only IL 13  [ **IL13** ]  but also IL 4 mediated signal transduction and Stat6 responsive gene expression.  Ref: 11861389 Cancer Res, 2002
- To elucidate the mechanism, we utilized four head and neck cancer cell lines SCC 25, HN12, KCCT873, and YCUM911, which express high levels of IL 13R  [ **IL13RA2** ] , and IL 13  [ **IL13** ]  toxin is highly cytotoxic to these cells.  Ref: 11862421 Cancer Immunol Immunother, 2002
